# Multiple Mutations and Overexpression in the *CYP51A* and *B* Genes Lead to Decreased Sensitivity of *Venturia effusa* to Tebuconazole

**DOI:** 10.3390/cimb44020047

**Published:** 2022-01-27

**Authors:** Logan C. Moore, Timothy B. Brenneman, Sumyya Waliullah, Clive H. Bock, Md Emran Ali

**Affiliations:** 1 Department of Plant Pathology, Coastal Plain Experiment Station, The University of Georgia, Tifton, GA 31793, USA; logan.moore26@uga.edu (L.C.M.); arachis@uga.edu (T.B.B.); sumyya.waliullah@uga.edu (S.W.); 2 United States Department of Agriculture-Agricultural Research Service Southeastern Fruit and Tree Nut Research Station, Byron, GA 31008, USA; clive.bock@usda.gov

**Keywords:** *V. effusa*, mutation, overexpression, pecan, DMI, resistance, fungicide

## Abstract

Multiple demethylation-inhibiting (DMI) fungicides are used to control pecan scab, caused by *Venturia effusa*. To compare the efficacy of various DMI fungicides on *V. effusa*, field trials were conducted at multiple locations applying fungicides to individual pecan terminals. In vitro assays were conducted to test the sensitivity of *V. effusa* isolates from multiple locations to various concentrations of tebuconazole. Both studies confirmed high levels of resistance to tebuconazole. To investigate the mechanism of resistance, two copies of the *CYP51* gene, *CYP51A* and *CYP51B*, of resistant and sensitive isolates were sequenced and scanned for mutations. In the *CYP51A* gene, mutation at codon 444 (G444D), and in the *CYP51B* gene, mutations at codon 357 (G357H) and 177 (I77T/I77L) were found in resistant isolates. Expression analysis of *CYP51A* and *CYP51B* revealed enhanced expression in the resistant isolates compared to the sensitive isolates. There were 3.0- and 1.9-fold increases in gene expression in the resistant isolates compared to the sensitive isolates for the *CYP51A* and *CYP51B* genes, respectively. Therefore, two potential mechanisms—multiple point mutations and gene over expression in the *CYP51* gene of *V. effusa* isolates—were revealed as likely reasons for the observed resistance in isolates of *V. effusa* to tebuconazole.

## 1. Introduction

Pecans are an important crop in the southeastern United States and are increasing in importance in other countries [1]. The U.S. produced 302 million pounds of in-shell pecans in 2020, and the state of Georgia is the second leading producer of pecans in the U.S., producing 64 million kilograms in 2020 [2]. Pecan scab is caused by the plant pathogenic fungus *Venturia effusa*, which thrives in conditions of high temperatures, high humidity, and frequent rainfall, which are common to the southeastern United States [3,4]. This disease is polycyclic, having multiple generations per year, and overwinters as lesions on twigs and shucks within the canopy of the tree. The lesions sporulate in the spring, and conidia are dispersed down through the canopy by rainfall, infecting the growing fruit and foliage [5]. The fungus has historically been classified as being strictly asexual; however, evidence of the sexual stage has been documented in the lab but has still not been found in the field [6,7]. Scab infections can result in significant yield losses to pecans if left unmanaged [8,9]. Although the most effective method for controlling *V. effusa* is to plant resistant cultivars, selection has resulted in the adaptation of the pathogen to be pathogenic on historically resistant cultivars [10,11,12,13,14,15]. Thus, frequent use of fungicides is the most widely adopted management approach for controlling scab in commercial pecan orchards in the Southeast. However, fungicide control of scab is one of the largest costs for pecan growers in the region. 

Demethylation-inhibiting (DMI) fungicides were first introduced for use against plant diseases in the 1970s and are now the most widely adopted and important group of fungistatic agents used worldwide both in medicine and in agriculture [16]. Since their discovery, over 30 DMI fungicides have been synthesized for use in agriculture [17]. They function by impeding sterol C-14 a-demethylation of 24-methylenedihydrolanosterol, which is a critical component in the formation of ergosterol in fungi. Ergosterol is vital to fungal cell membranes and regulates membrane permeability and fluidity, which is why the enzymes that synthesize ergosterol are important targets for anti-fungal agents [18]. Since the DMIs have been so widely used and repeatedly applied to large areas of crops year after year, there have been many reports of resistance in fungi, beginning in the 1980s, only a decade after their release [19,20,21]. DMI fungicides are single-site, broad-spectrum fungicides that are used for the pre- and post-infection control of various pathogens [22]. The development of resistance to the DMI fungicides has led to the decreased sensitivity of various pathogens to a range of DMI fungicides, including the apple scab pathogen *Venturia inaequalis*, a close relative to the pecan scab pathogen, *V. effusa* [23]. DMI fungicides were first labeled for use against *V. effusa* in 1988 and are heavily used to combat scab each year [24]. The DMIs labeled for use in pecan include difenoconazole, propiconazole, fenbuconazole, tetraconazole, metconazole, flutriafol, mefentrifluconazole, and tebuconazole. Isolates of *V. effusa* collected in 2003 were found to be less sensitive to propiconazole when compared to the baseline isolates reported in 1997 [25,26]. Standish et al. [27] reported the field resistance of *V. effusa* to tebuconazole and confirmed the reduced sensitivity using an in vitro assay. In a separate study, Standish et al. [28] demonstrated insensitivity to tebuconazole to be phenotypically stable. Luckily, there are fungicides belonging to several other fungicide classes (Fungicide Resistance Action Committee [FRAC] Codes: 3, 11 U12, P7, 30, 1, M03, and 7) that are labelled for use on pecan to help mitigate *V. effusa* [29].

To date, resistance to DMI fungicides has been reported in 37 fungal species. Cross resistance has been documented among DMI fungicides used against the same pathogen [30,31,32,33]. Cross resistance has been observed in *V. effusa* among tebuconazole, fenbuconazole, and propiconazole, but has not yet been found for difenoconazole, which is widely applied to control scab [25,26]. Resistance to DMIs is typically caused by amino acid changes in the *CYP51* gene, overexpression of the *CYP51* gene, or by efflux pumps reducing intracellular fungicide accumulation within the pathogen [18,34,35]. Tucker et al. [36] investigated the mechanism of resistance to DMI fungicides in the pathogen *Blumeria graminis* and found five separate amino acid substitutions in the *CYP51B* target gene, four of which were novel, showing that multiple mutations may confer resistance to DMI fungicides. *V. inaequalis*, a close relative of *V. effusa*, also displays resistance to the DMI fungicides. Villani et al. [37] investigated the mechanisms of DMI resistance in *V. inaequalis* and found overexpression of the *CYP51A1* gene in resistant isolates compared to the sensitive isolates. Hayashi et al. [38] demonstrated that ABC transporters can lead to the decreased sensitivity of *Botrytis cinerea* to DMI fungicides. The results from these studies show that several potential factors may be contributing to the resistance of *V. effusa* to the DMI fungicides. 

The fungicide resistance studies describing resistance in *V. effusa* to the DMIs to date have reported only phenotypic data, and the specific mechanism of resistance remains unknown [26,27,39]. The goal of the current study was to determine the mechanism of resistance of *V. effusa* to the DMI tebuconazole to provide further insights regarding DMI insensitivity prevalence in commercial pecan orchards in the southeastern U.S. 

## 2. Results

### 2.1. Evaluation of Field Sensitivity of V. effusa to DMI Fungicides 

**Figure 1:**
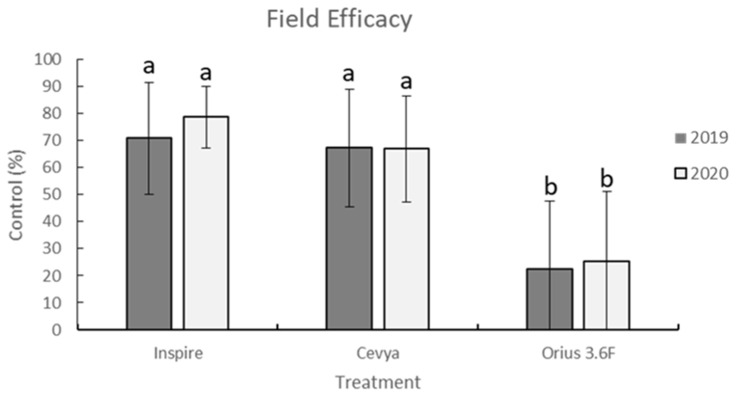
Field efficacy (percent reduction in severity of symptoms of scab (caused by *Venturia effusa* compared to the control) of Inspire (difenoconazole), Cevya (mefentrifluconazole), and Orius 3.6F (tebuconazole). Different letters indicate statistical differences based on the Tukey-Kramer mean separation procedure. Error bars on the graph represent standard deviations of the mean.

The field experiment showed variability in scab pressure among locations, with the nontreated terminals at different locations having scab severity ranging from 0 to 100% in 2019 and 2020 (Table 1). Repeated applications of several DMI fungicides including Inspire resulted in 70.9 and 78.7% control, Cevya led to 67.3 and 67.0% control, and Orius 3.6F led to 22.4 and 25.2% control in 2019 and 2020, respectively. At most locations, the scab control efficacy of Inspire and Cevya were statistically similar, while the efficacy of Orius 3.6F was significantly less than that achieved by the application of either Inspire or Cevya (Figure 1; Table 1). 

### 2.2. Determination of In Vitro Sensitivity of V. effusa to Tebuconazole

Results from the in vitro assay of isolates from the different locations revealed variability in sensitivity to tebuconazole. At 1 µg/mL tebuconazole, the relative growth values (RGr) ranged from 48 to 173%, at 3 µg/mL RGr ranged from 23 to 133%, and at 10 µg/mL the RGr ranged from 3 to 86% (Table 2). While variability was evident, all locations presented high levels of resistance to tebuconazole (Table 2). Among the 11 isolates of *V. effusa* that were used to determine the mechanism of resistance to tebuconazole, the highly resistant isolates grew well on tebuconazole amended media containing 10 µg/mL tebuconazole, with RGr values ranging from 58% to 110% at 10 µg/mL tebuconazole, and a mean RGr value of 71% (Table 3). Since resistant isolates of fungal pathogens may be defined as having 50% or higher RGr in the presence of the discriminatory concentration (1 µg/mL tebuconazole), the resistant isolates with over 50% growth on 10 µg/mL tebuconazole confirm that the isolates are highly resistant to tebuconazole [39]. All sensitive isolates (from 1993) were completely suppressed (0% RGr) by 1 µg/mL tebuconazole (Table 3). The assay confirmed that the sensitive isolates were highly sensitive to tebuconazole, while the resistant isolates were highly resistant to tebuconazole.

### 2.3. Sequence Analysis of CYP51A and B Revealed Mutations among Resistant Isolates

Several nucleotide anomalies were observed in both the *CYP51A* and *B* genes; however, most of the single nucleotide polymorphisms were silent mutations that did not lead to a change in the translated amino acid sequence. In the *CYP51A* gene, there was one mutation (the G444D mutation) that did impact the translated amino acid sequence. The G444D mutation was present in four out of the eight resistant isolates but not in any of the sensitive isolates. The SNPs in sequences resulted in an amino acid switch from Glycine to Aspartate at location 444. In the *CYP51B* gene, amino acid mutations were present at two locations. The I77T mutation was present in five of the resistant isolates and none of the sensitive isolates and occurred as a result of the single nucleotide base substitutions of thymine to cytosine at nucleotide location 230, resulting in the amino acid change of Isoleucine to Threonine at location 77. The I77L mutation occurred in one resistant isolate and occurred as a result of a single nucleotide base substitution of thymine to adenine at nucleotide location 230, resulting in an amino acid change of Isoleucine to Leucine at location 77. The G357H mutation was present in six of the eight resistant isolates and none of the sensitive isolates and occurred as a result of a single nucleotide base substitution of adenine to thymine at nucleotide location 1071, resulting in an amino acid change of Glycine to Histidine at location 357.

### 2.4. Higher Gene Expression among the Resistant Isolates

**Figure 2:**
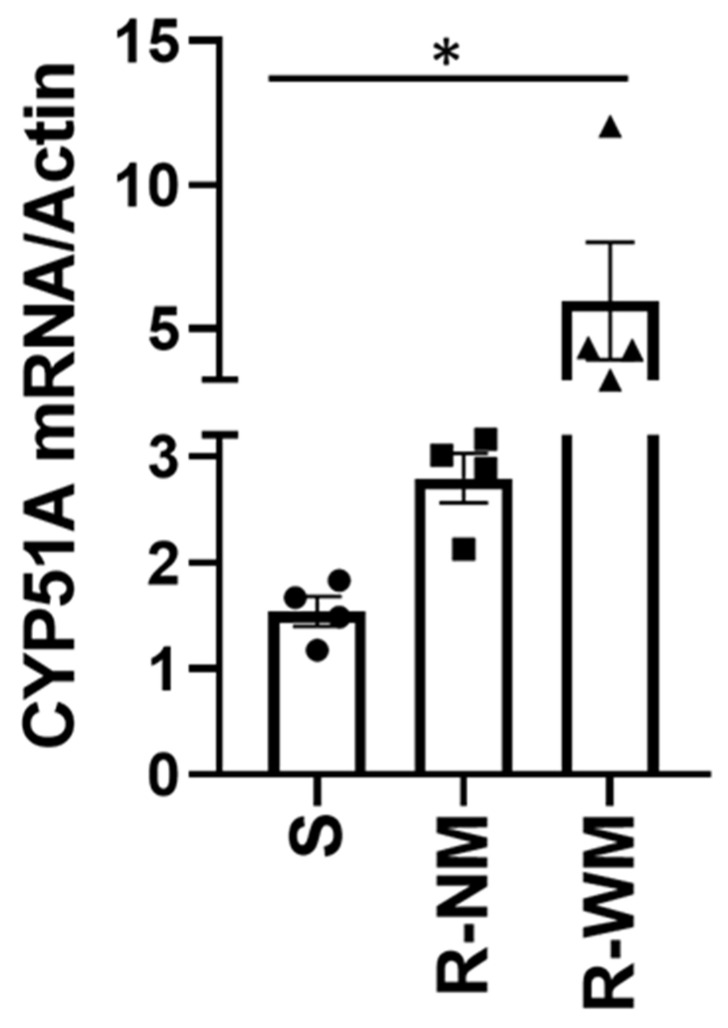
Relative expression of the *CYP51A* gene in *Venturia effusa*. Asterix (*) indicates a statistical difference between resistant isolates with the G444D mutation and sensitive groups (*p* = 0.0283). The “S” on the x-axis indicates the 4 sensitive isolates, while the “R-NM” represents the 4 resistant isolates without the G444D mutation, and the “R-WM” represents the 4 resistant isolates with the G444D mutation. The black circles represent individual sensitive isolates, while the black triangles and squares represent the resistant isolates with and without the G444D mutation, respectively. Error bars represent standard deviation from the mean.

**Figure 3:**
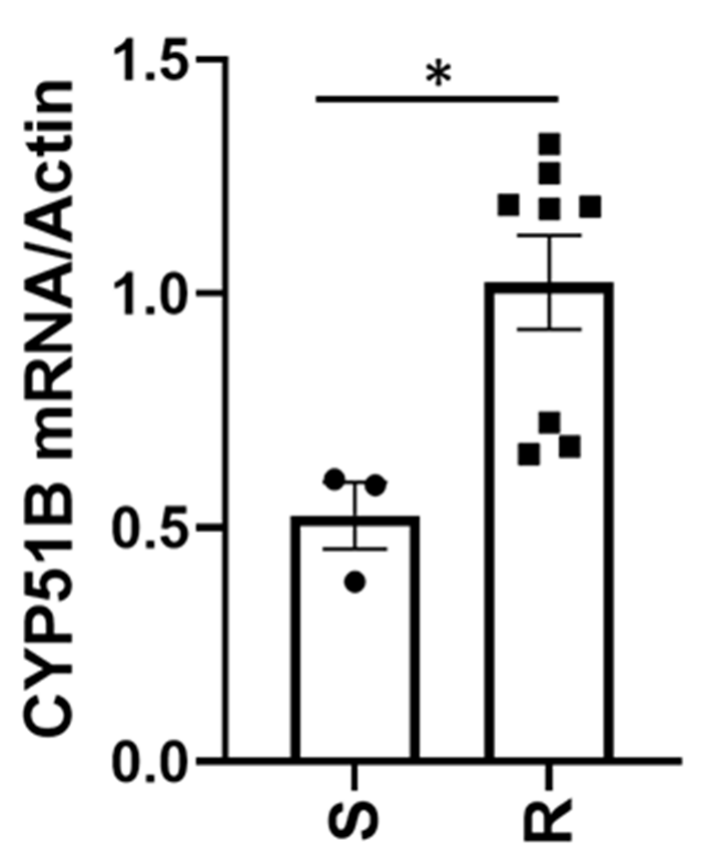
Relative expression of the *CYP51B* gene in *Venturia effusa*. Asterix (*) indicates statistical differences between resistant and sensitive groups (*p* = 0.0189). The “S” on the x-axis indicates the 3 sensitive isolates, while the “R” represents the 8 resistant isolates. The black circles indicate individual sensitive isolates, while the black squares indicate individual resistant isolates. Error bars represent standard deviation from the mean.

Relative expression (RE) analysis revealed that resistant isolates expressed both *CYP51A* and *CYP51B* genes more than the sensitive isolates. This analysis revealed that the resistant isolates with the G444D mutation expressed the *CYP51A* gene more compared to the resistant isolates that did not contain the mutation. The sensitive isolates mean RE was 1.48, whereas the resistant isolates with the G444D mutation had a mean RE value of 5.96, and the resistant isolates without the G444D mutation had a mean RE value of 2.80 (Figure 2). With the *CYP51B* gene, sensitive isolates had a lower RE compared to the resistant isolates. Sensitive isolates had a mean RE value of 0.53, while the resistant isolates had a mean RE value of 1.02 (Figure 3).

## 3. Discussion

Our results indicate that difenoconazole and mefentrifluconazole are both highly active on *V. effusa*, while tebuconazole is not, due to the presence of resistant isolates. Confirmation of reduced sensitivity led to the collection of resistant isolates and investigation of the exact mechanism of resistance. Although some possible mechanisms of resistance in *V. effusa* to the DMI fungicides are presented, definitive conclusions regarding the mechanism of resistance should not be made. We suggest two mechanisms based on the results of this study, including novel mutations in both the *CYP51A* and *B* genes (G444D, G357H, I77T, and I77L) (Table 4), as well as 3.0- and 1.9-fold increases in expression of the *CYP51A* and *B* genes, respectively (Figure 2 and Figure 3). These are novel mutations and have not been previously described for resistance to fungicidal compounds. These same mutations, as well as overexpression may also be responsible for the resistance of *V. effusa* observed to fenbuconazole and propiconazole [25,26,40]. Mutations in the *CYP51* gene are a common cause of resistance to DMIs and have been found in several other pathogens [36,41,42]. Overexpression of the *CYP51* genes is also commonly found in resistant isolates of various pathogens [43,44,45]. We anticipated either mutations or overexpression of the *CYP51* gene to be the cause of the resistance, and we found mutations and overexpression to be present in DMI-resistant isolates of *V. effusa*. The resulting amino acid changes and overexpression in isolates of a single pathogen species exhibiting resistance to the DMI fungicides is also not uncommon [46,47]. While this study did not address ABC transporters and other efflux transporters, it is possible that they may also play a role in resistance to the DMI fungicides in *V. effusa*, and future studies should be aimed at investigating ABC transporters and other efflux transporters as potential sources of DMI resistance. 

The DMI fungicides are used heavily in commercial pecan orchards to control *V. effusa*. Understanding fungicide sensitivity in *V. effusa* can aide in the further development and strengthening of fungicide resistance management rotation programs. The results help outline the necessity for further fungicide research and development required to control *V. effusa* efficiently and sustainably. Difenoconazole is the most widely used DMI fungicide in commercial pecan orchards and is labeled for use on pecan only as a mixture combined with other active ingredients. The popular combination products are Amistar Top (difenoconazole + azoxystrobin) and Miravis Top (difenoconazole + pydiflumetofen). Combination products with more than one active ingredient in different fungicide classes can increase disease control and contribute to delaying fungicide resistance in certain pathogens. However, if one of the active ingredients in the premixture begins to lose its efficacy due to resistance development in the pathogen, the other active ingredient in the combination is more at risk of resistance development. So far, no resistance of *V. effusa* to difenoconazole has been reported, and combination products containing difenoconazole remain popular among commercial pecan growers. However, there is currently no baseline sensitivity data pertaining to *V. effusa* sensitivity to difenoconazole; therefore, slight shifts in sensitivity may go unnoticed and cannot be confirmed in the lab. Although resistance to difenoconazole has not been reported for *V. effusa*, it has been reported for several other pathogens, including *V. inaequalis*, *Lasiodiplodia theobromae*, *Penicillium* spp., *Botrytis cinereal*, *Alternaria* spp., *Aspergillus fumigatus*, etc. [36,45,48,49,50,51,52].

Mefentrifluconazole was recently labeled for use on pecan (Cevya), and although resistance has not yet been reported in *V. effusa*, resistance has already been detected in other pathosystems [33]. Not only has resistance to mefentrifluconazole been detected in other pathosystems, but cross-resistance between mefentrifluconazole and tebuconazole, as well as mefentrifluconazole and difenoconazole has been shown in other pathosystems [33,53,54]. Mefentrifluconazole is not yet widely used in commercial pecan orchards but may play a role in future fungicide rotation programs. However, since Cevya is a stand-alone DMI fungicide that runs a high risk of resistance development, its use should be limited, and it should only be applied in strict rotation and never applied alone in consecutive applications. Tebuconazole products are no longer widely used among commercial pecan growers due to very low activity as a result of fungicide resistance developing in *V. effusa*, as confirmed by our field and in vitro studies. Our results indicate that resistance of *V. effusa* to tebuconazole is widespread, and that the efficacy of tebuconazole across southern Georgia is low (Figure 1; Table 1 and Table 2). Mefentrifluconazole and difenoconazole are both newer DMI fungicides and are active on scab even when other DMI fungicides are not. Newer DMI fungicides maintaining efficacy while older DMI fungicides fall victim to resistance development is not a novel observation [33,55]. It has been proposed that the high structural flexibility of the mefentrifluconazole molecule is to blame for the limited cross resistance being observed [56]. Because of flexible isopropanol linkers, mefentrifluconazole molecules are able to settle into the binding pocket of the *CYP51* enzyme, resulting in strong inhibition of enzymatic activity, even when target site alterations due to amino acid substitutions may be present [33,56].

The goal of determining the mechanism of resistance is to both add to our knowledge regarding resistance development in a pathogen, as well as providing a basis for developing detection methods that can be used rapidly to identify and track the specific resistance trait in orchard populations of *V. effusa* in the future. These rapid detection methods are not uncommon and have been proven effective in various other pathogens in regard to resistance to the DMIs, as well as other fungicide groups [57,58]. Since *V. effusa* is a very slow-growing pathogen, taking approximately 30 days for a colony to grow to a diameter of 25 mm, a rapid method for detecting resistance to DMI (and other) fungicides would be a valuable tool to provide to growers and other stakeholders to better characterize the pathogen population present in their orchard, which ultimately will help optimize management of the populations of *V. effusa* to minimize the risk of further development and spread of fungicide resistance, and improve the efficacy and sustainability of scab control. 

## 4. Materials and Methods

### 4.1. Evaluation of Field Sensitivity of V. effusa to DMI Fungicides

In 2019 and 2020, 11 pecan orchards were selected in Tift, Berrien, Wilcox, Lanier, Crisp, Dougherty, and Sumter counties in Georgia. The orchards were chosen as they were planted in scab-susceptible cultivars including Cunard, Desirable, Pawnee, and Wichita. The orchards varied in tree spacing, age, disease pressure, and environmental conditions. At each location, 8 consecutive trees within a row received no commercial fungicide applications from nut set to harvest. The experimental design was a randomized complete block design at each location, with each tree being a block with one replicate of each of the 4 treatments. Thus, the treatments were applied directly to individual fruiting terminals on the pecan trees that were flagged with different colored ribbons to indicate which treatment the terminal received. Each terminal contained one or more clusters of pecans. The treatments were applied by spraying to initial runoff using a handheld sprayer (Project Source model #5318). The treatments were as follows: Orius 3.6F (tebuconazole; Makhteshim Agan of North America, Inc. Raleigh, NC, USA) at 584.6 milliliters per hectare (mL/ha), Inspire (difenoconazole; Syngenta Crop Protection, Greensboro, NC, USA) applied at 489.6 mL/ha, Cevya (mefentrifluconazole; BASF Corporation, Research Triangle Park, NC, USA) applied at 365.4 mL/ha, and an untreated control (Table 5). The terminals received the same application on a 14-day schedule from bud break to shell hardening for a total of 7 applications in 2019 and 8 applications in 2020. The severity of scab symptoms was estimated after shell hardening on each of the fruit on each terminal by visual observation using a 0–100% rating scale. Relative control (%) for each treatment was calculated based on the severity on the nontreated control. 

### 4.2. Determination of In Vitro Sensitivity of V. effusa to Tebuconazole

At each location in both 2019 and 2020, leaf and nut scab samples were taken in July from multiple untreated trees and were placed in Ziploc bags containing a wet paper towel to help induce sporulation. After 24 h, the samples were used to conduct an in vitro sensitivity assay to tebuconazole, following the procedure described by Seyran et al. [39]. There were two plates (repetitions), with three groups of conidia from different scab lesions per plate. Each group contained a conidial slurry obtained from 15 distinct *V. effusa* lesions, and antibiotics were added to the solution to prevent contamination. The concentrations of tebuconazole in the media were 0, 1, 3, and 10 µg/mL, prepared by serial dilution from technical grade tebuconazole (Chem Service Inc., West Chester, PA, USA; 98.1% purity). Conidia that germinated and grew on media amended with 10 µg/mL tebuconazole were considered to be highly resistant and were removed from the fungicide amended media under a dissecting microscope using a sterile needle and were plated as monoconidial isolates on non-amended potato dextrose agar (PDA) in Petri plates to be used for later molecular analysis to determine the mechanism of tebuconazole resistance. The monoconidial isolates of *V. effusa* that were considered to be sensitive to tebuconazole were isolated from two baseline orchards in 1993 and 1994, stored in sterile water in 1996, and were revived in 2020 by culture on PDA. The isolates were collected for a baseline sensitivity study in *V. effusa* to the DMIs propiconazole and fenbuconazole in Troup and Jeff Davis counties in Georgia and have never been exposed to modern fungicides [25]. Additional mycelial growth assays were performed to confirm the sensitivity of these isolates to tebuconazole. The resistant isolates were from orchards in Berrien and Dougherty counties in Georgia, collected in 2020. All monoconidial isolates cultured on PDA were incubated in the dark at 25 °C for 4 weeks to reach acceptable colony size (15–25 mm in diameter). A plug of PDA with mycelium of *V. effusa* was taken from each colony using a 4 mm cork borer and was homogenized in 1 mL sterile water in a microfuge tube using a bead beater for 20 s. Tebuconazole amended media was prepared in Petri plates containing 0, 1, 3, and 10 µg/mL tebuconazole exactly as described before with the previous assay. Using a 4 mm cork borer, two 4 mm wells were prepared near the center of each plate of the fungicide amended media, approximately 30 mm apart, and 20 uL of the mycelial slurries were pipetted into the wells. The Petri plates were incubated in the dark at 25 °C for 4 weeks, and the diameters of the colonies were measured using a ruler. 

### 4.3. Investigating Mechanisms of Resistance

#### 4.3.1. DNA Extraction

All monoconidial isolates were grown on PDA amended with antibiotics (50 mg/L each of streptomycin, rifampin, and chloramphenicol) prior to DNA extraction. The isolates were incubated in the dark for 4 weeks at 25 °C prior to extraction. After 4 weeks, the colonies had reached sufficient diameter (15–25 mm), and 50 to 100 mg of mycelium was collected from the surface of the agar using a scalpel. An aliquot of 500 µL lysis buffer (Norgen DNA Isolation Kit; Norgen Biotek Corp., Thorold, ON, Canada) and fifteen to twenty small glass beads were added to a 1.5 mL safe-lock tube (Eppendorf Canada Ltd., Mississauga, ON, Canada), and using a FastPrep FP 120 cell distributor (Qbiogene, Carlsbad, CA, USA) were homogenized twice at a speed 4.0 for 30 s each. After lysis, the DNA was extracted using an UltraClean Microbial DNA Isolation Kit (Qiagen, Germantown, MD, USA) following the manufacturer’s protocol. The purity and quantity of DNA were measured with a NanoDrop spectrophotometer (Nanaodrop Lite, Thermo Scientific, Waltham, MA, USA). A polymerase chain reaction (PCR) was used to amplify the DNA of both the *CYP51A* and *CYP51B* genes using the SYBR Green PCR Master Mix (Thermo Fisher Scientific Inc, Waltham, MA, USA) with the primers listed above (Table 2). PCR conditions varied for each primer set that was used to amplify different fragments of the *CYP51* genes. For the sequencing of the *CYP51* genes, DNA was extracted from 3 sensitive isolates and 8 resistant isolates using the protocol described above. 

#### 4.3.2. Primer Design

**Figure 4:**
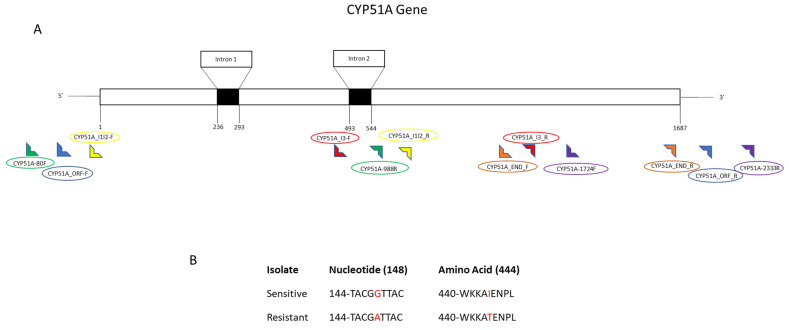
Schematic of the *CYP51A* gene and the detected mutation in *Venturia effusa*. (**A**) Exon and intron organization of *CYP51A*. Primers are indicated by 90° symbols at the bottom of the schematic, and primer names are listed above or below the primer symbol. Each primer set contains the same color symbol and circle surrounding the name of the primer. (**B**) Characteristics of sensitive and resistant isolates comparing nucleotide and amino acid mutations indicated with a red nucleotide or amino acid identifier. The mutation described here is the G444D amino acid substitution.

**Figure 5:**
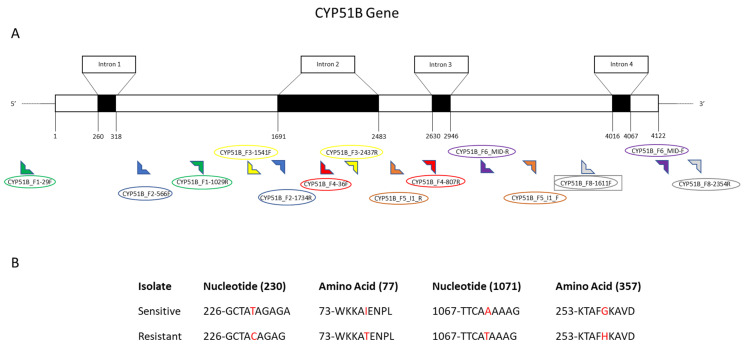
Schematic of the *CYP51B* gene and the detected mutations in *Venturia effusa*. (**A**) Exon and intron organization of *CYP51B*. Primers are indicated by 90° symbols at the bottom of the schematic, and primer names are listed above or below the primer symbol. Each primer set contains the same color symbol and circle surrounding the name of the primer. (**B**) Characteristics of sensitive and resistant isolates comparing nucleotide and amino acid mutations indicated with a red nucleotide or amino acid identifier. The mutations described here are the I77T/L and G357H amino acid substitutions.

Six sets of primers were designed to sequence the *CYP51A* gene including the coding region based on the sequence obtained from GenBank (Table 6). The *V. effusa* albino strain chromosome 1 sequence with the accession number CP042185.1 was used as a reference sequence [59]. The 1686 bp sequence of the *CYP51A* gene on chromosome 1 was at 371,163 to 372,848 bp. The primer pairs CYP51A_I1I2_F1/CYP51A_I1I2_R1, CYP51A_I3_F1/CYP51A_I3_R1 and CYP51A_End_F1/CYP51A_End_R1 were designed to amplify a 1578 bp fragment of the coding region. The primer pair CYP51A_P1-80 F/CYP51A_P1-988 R were developed to amplify part of the *CYP51A* coding region and the upstream 368 bp of promoter regions at the 5′ end based on the GenBank accession number CP042185.1 (370,698 bp to 373,113 bp). A 610 bp fragment including 198 bp downstream of the 3′ end of the *CYP51A* gene was amplified using the primer set CYP51A_P2-1724 F/CYP51A_P2-2333 R. The primer set CYP51A_ORF_F1/CYP51A_ORF_R1 was developed to amplify the whole coding region at once using conventional PCR for subsequent sequencing of the gene (Table 6, Figure 4). Similarly, for the sequencing of the *CYP51B* gene, 8 sets of primer pairs were designed based on the sequence of *V. effusa* albino strain chromosome 5 obtained from NCBI GenBank [59]. The GenBank accession number used as the reference for the sequence is CP042189.1, with a position from 1,774,471 to 1,778,656 bp that covers the whole *CYP51B* gene. The primer pair CYP51B_F1-29 F/CYP51B_F1-1029 R set was designed to amplify an amplicon of 1001 bp including a part of the upstream promoter region (~50 bp) and a part of the coding region (847 bp) with one intron region. The primer set CYP51B_F2-566 F/CYP51B_F2-1734 R was designed to sequence the 1173 bp of the coding region. The CYP51B_F3-1541F/ CYP51B_F3-2437R, and CYP51B_F4-36F/CYP51B_F4-807R primer sets were designed to amplify fragments of the coding region including the second intron site. The remaining three sets of primers including CYP51B_F5_I1_F/CYP51B_F5_I1_R, CYP51B_F6_Mid_F/CYP51B_F6_Mid_R and CYP51B_F7_I2_F/CYP51B_F7_I2_R were developed to amplify the coding region including the third and fourth intron sites to amplify the remaining portion of the *CYP51B* coding region, totaling 4123 bp. The primer set CYP51B_F8-1611 F/CYP51B_F8-2354 R amplified a 744 bp fragment including 284 bp downstream of the 3′ end of the *CYP51B* gene for the sequencing of the terminator region of the gene (Table 6, Figure 5).

#### 4.3.3. Sequencing and Analysis of the *CYP51A* and *B* Sequences

PCR products were separated by gel electrophoresis on a 1% agarose gel (BioRad, Hercules, CA, USA) stained with GelRed (Biotium, Fremont, CA, USA) and run in 1X Tris/Borate/EDTA buffer at 90 V for 40 min. Images of the gel were captured on a UV Geldoc gel imager (Analyik Jena, Upland, CA, USA). DNA was purified using the Quantum Prep PCR Kleen Spin purification kit (BioRad, Hercules, CA, USA) using the protocol provided by the manufacturer. Purified DNA was sent to Retrogen Inc. (San Diego, CA, USA) for Sanger sequencing with both forward and reverse internal primers for each primer set. Once sequencing results were obtained, introns were removed, the DNA was aligned, translated to amino acid sequence, and screened for possible mutations using Geneious Prime software V 2019.2.3 (https://genious.com, accessed on 15 April 2021). 

#### 4.3.4. *CYP51A* and *B* Gene Expression

All isolates were grown on PDA amended with antibiotics (50 mg/L each of streptomycin, rifampin and chloramphenicol) prior to RNA extraction. RNA was extracted from each isolate using the RNeasy kit (Qiagen, Germantown, MD, USA) following the manufacturer’s protocol. Synthesis of cDNA from the extracted RNA was achieved using the iScript^TM^ cDNA synthesis kit (BioRad, Hercules, CA, USA) following the manufacturer’s protocol. Expression analysis was conducted through a real-time quantitative PCR (qPCR) assay using a BioRad CFX connect real-time system (BioRad, Hercules, CA, USA) to quantify the expression of *CYP51A* and *B* genes from all the isolates using the primers listed in (Table 1). The SsoAdvanced™ Universal SYBR^®^ Green Supermix (Bio-Rad Inc., Hercules, CA, USA) was used for qPCR analysis. The total reaction volume was 10 μL containing 5 μL of SYBR^®^ Green Supermix, 0.4 μL of 1000 nM of each forward and reverse primers (Table 2), and 2 μL (10 pg) of cDNA; the balance of volume was made up with molecular grade water. The PCR protocol for the expression study had an annealing/extension temperature of 60 °C, and a melt curve analysis was included. The CFX Maestro™ Software (Bio-Rad Inc., Hercules, CA, USA) was used to analyze all qPCR data. Relative gene expression was calculated as the ratio between *CYP51A* and *B* and the reference control gene, β-actin, following the 2^−ΔΔCt^ equation [60].

### 4.4. Data Analysis

For the field experiment data comparing DMI fungicide treatments, the univariate procedure was used to confirm that data were normally distributed. Data were analyzed using a generalized linear mixed model (PROC GLIMMIX) in SAS V9.4 (SAS Institute, Cary, NC), with block, cultivar, and year as random effects, and location and treatment as fixed effects. Location was treated as a fixed effect due to a significant interaction of treatment*location (Table 7). Locations containing little (<10% scab severity) to no disease pressure on untreated terminals were excluded from the dataset. The 95% confidence intervals of the treatment means were calculated. A Tukey-Kramer test was conducted as the mean separation procedure (α = 0.05).

The qPCR *CYP51A* and *B* expression data are presented as mean ± the standard error of the mean (SEM). Graph preparations and analysis of the data were performed using GraphPad Prism 8 software (GraphPad Software, Inc. San Diego, CA, USA). Data were subjected to a mean separation procedure using a two-tailed Student *t*-test at *p* < 0.05.

## Figures and Tables

**Table 1:** Percent scab control comparing different DMI products and active ingredients in field trials at each of 11 locations in 2019 and 2020. Means with different letters are significantly different at each location for each year based on the Tukey-Kramer mean separation procedure. The “-” symbol indicates missing data due to lack of scab pressure at that location in that year.

Location	County	Treatment	2019 % Control	2020 % Control
**N**	Dougherty	Orius 3.6F	2.7 B	49.7 B
		Inspire	50.8 A	81.6 A
		Cevya	56.5 A	80.8 A
**B3**	Dougherty	Orius 3.6F	30.8 A	46.5 A
		Inspire	66.4 A	82.8 A
		Cevya	63.7 A	44.3 A
**JB**	Sumter	Orius 3.6F	51.3 A	45.2 B
		Inspire	76.5 A	88.2 A
		Cevya	75.3 A	81.4 A
**PKD**	Dougherty	Orius 3.6F	3.8 B	6.9 B
		Inspire	58.2 A	63.0 A
		Cevya	48.5 A	64.8 A
**JB2**	Crisp	Orius 3.6F	52.5 B	-
		Inspire	80.3 AB	-
		Cevya	89.7 A	-
**SH**	Wilcox	Orius 3.6F	57.1 B	-
		Inspire	97.2 A	-
		Cevya	81.4 AB	-
**BP**	Berrien	Orius 3.6F	14.6 B	39.1 B
		Inspire	71.1 A	81.0 A
		Cevya	57.7 A	65.5 A
**JD**	Lanier	Orius 3.6F	48.3 B	77.6 A
		Inspire	81.7 A	92.7 A
		Cevya	80.5 A	88.0 A
**CR**	Berrien	Orius 3.6F	55.7 A	9.5 B
		Inspire	89.5 A	75.0 A
		Cevya	75.0 A	62.7 A
**PW**	Tift	Orius 3.6F	9.8 B	12.9 B
		Inspire	82.0 A	84.4 A
		Cevya	81.2 A	76.4 A
**PD**	Tift	Orius 3.6F	49.8 B	29.8 B
		Inspire	87.2 A	85.5 A
		Cevya	82.2 A	73.5 A

**Table 2:** Results from the rapid assays to determine *Venturia effusa* sensitivity to tebuconazole in 2019 and 2020 from the 11 locations where the field experiments were conducted in southern Georgia. Relative growth (RGr) values are presented in the far-right columns for both years.

Location	County	Concentration	2019 RGr	2020 RGr
**N**	Dougherty	1 µg/mL	100%	100%
		3 µg/mL	100%	61%
		10 µg/mL	86%	16%
**B3**	Dougherty	1 µg/mL	100%	56%
		3 µg/mL	100%	54%
		10 µg/mL	44%	5%
**JB**	Sumter	1 µg/mL	100%	97%
		3 µg/mL	85%	46%
		10 µg/mL	25%	9%
**PKD**	Dougherty	1 µg/mL	49%	48%
		3 µg/mL	46%	57%
		10 µg/mL	19%	21%
**JB2**	Crisp	1 µg/mL	48%	27%
		3 µg/mL	23%	9%
		10 µg/mL	3%	0%
**SH**	Wilcox	1 µg/mL	100%	N/A
		3 µg/mL	87%	N/A
		10 µg/mL	36%	N/A
**BP**	Berrien	1 µg/mL	1%	72%
		3 µg/mL	58%	32%
		10 µg/mL	25%	10%
**JD**	Lanier	1 µg/mL	25%	3%
		3 µg/mL	11%	0%
		10 µg/mL	1%	0%
**CR**	Berrien	1 µg/mL	N/A	74%
		3 µg/mL	N/A	58%
		10 µg/mL	N/A	8%
**PW**	Tift	1 µg/mL	67%	82%
		3 µg/mL	43%	51%
		10 µg/mL	3%	12%
**PD**	Tift	1 µg/mL	76%	66%
		3 µg/mL	63%	23%
		10 µg/mL	15%	4%

**Table 3:** Sensitivity of the isolates of *Venturia effusa* used to determine the mechanism of resistance to tebuconazole at 1, 3, and 10 µg/mL.

Isolate Name	Georgia County	Sensitivity Status	RGr at 1 µg/mL	RGr at 3 µg/mL	RGr at 10 µg/mL
**T11**	Troup	Sensitive	0%	0%	0%
**T15**	Troup	Sensitive	0%	0%	0%
**T37**	Troup	Sensitive	0%	0%	0%
**108**	Berrien	Resistant	100%	98%	100%
**241**	Berrien	Resistant	100%	73%	58%
**253**	Berrien	Resistant	100%	73%	58%
**254**	Berrien	Resistant	92%	100%	67%
**407**	Dougherty	Resistant	100%	76%	67%
**410**	Dougherty	Resistant	100%	85%	62%
**482**	Dougherty	Resistant	97%	75%	66%
**803**	Dougherty	Resistant	100%	77%	77%

**Table 4:** The isolates of *Venturia effusa* used to determine the mechanism of resistance to tebuconazole, and the mutations (G444D (the G444D mutation represents a glycine to aspartic acid amino acid substitution), I77T (the I77T mutation represents an isoleucine to threonine amino acid substitution), I77L (the I77L mutation represents an isoleucine to leucine amino acid substitution), and G357H (the G357H mutation represents a glycine to histidine amino acid substitution)) that were observed in the resistant isolates.

Sensitivity	Isolate Name	*CYP51A* Gene	*CYP51B* Gene	RGr at 10 µg/mL
**Sensitive**	T11	None	None	0%
	T15	None	None	0%
	T37	None	None	0%
**Resistant**	407	G444D	I77T and G357H	67%
	410	G444D	I77T and G357H	62%
	482	G444D	I77T and G357H	66%
	241	None	I77L and G357H	58%
	253	None	I77T and G357H	58%
	254	None	I77T and G357H	67%
	803	G444D	None	77%
	108	None	G357H	100%

**Table 5:** List of demethylation inhibitor fungicide products and active ingredients compared for efficacy for controlling scab (caused by *Venturia effusa*) on pecan in field trials conducted in 2019 and 2020 in southern Georgia.

Fungicide Product	Active Ingredient	Rate/ha
**Orius 3.6F**	Tebuconazole	584.6 mL
**Inspire**	Difenoconazole	489.6 mL
**Cevya**	Mefentrifluconazole	365.4 mL
**Nontreated**	N/A	N/A

**Table 6:** The primers designed in this study for amplifying the *CYP51A* and *CYP51B* genes of *Venturia effusa*, and the primers designed for RT-qPCR *CYP51A* and *CYP51B* gene expression assay.

Purpose	Primer Name	Primer Sequence (5′-3′)	Gene	Size (bp)
Sequencing	CYP51A_ORF_F1	AATGGAAGGGTCCTCGCATG	*CYP51A*	2009
	CYP51A_ORF_R1	AGTTCGAAGCCGCCTAGAAC	*CYP51A*	
	CYP51A_I1I2_F1	CAGGCTACAATTCTGCCGC	*CYP51A*	674
	CYP51A_I1I2_R1	TGGATGAGGGTGACATAGGA	*CYP51A*	
	CYP51A_I3_F1	CGGTTCCGACGTCGTCTATG	*CYP51A*	687
	CYP51A_I3_R1	AGACACGAAGCTGTTCCTGG	*CYP51A*	
	CYP51A_End_F1	TACCTCGTCCTGGATCCTCC	*CYP51A*	605
	CYP51A_End_R1	GAGAGGTCCGGAGAAGAGGG	*CYP51A*	
	CYP51A_P1-80 F	TTGACTTGGATGTTGAGGCG	*CYP51A*	909
	CYP51A_P1-988 R	TGGAAGTCAGCGTATGTTCC	*CYP51A*	
	CYP51A_P2-1724 F	TATTCCCACTTCGCACATCC	*CYP51A*	610
	CYP51A_P2-2333 R	AGTCTTCCTTGGTCTACTTCG	*CYP51A*	
	CYP51B_F1-29 F	AGCCAACTGGTGAGATACGAC	*CYP51B*	1001
	CYP51B_F1-1029 R	TCGAAGAGGAGACAACGGG	*CYP51B*	
	CYP51B_F2-566 F	AGTCCGGAGCTACAATTGCC	*CYP51B*	1169
	CYP51B_F2-1734 R	AACAGGCACCTTTCCCTCAC	*CYP51B*	
	CYP51B_F3-1541 F	AATACGACGTACTCATCCTCCC	*CYP51B*	897
	CYP51B_F3-2437 R	TTGGTTCCTGAGCGTGTCAC	*CYP51B*	
	CYP51B_F4-36 F	ATTGCGCAAATTCGATCGG	*CYP51B*	772
	CYP51B_F4-807 R	AATCGTAAACCACTCCCTCG	*CYP51B*	
	CYP51B_F5_I1_F	TTTCCATGACCTTTTGCGCG	*CYP51B*	640
	CYP51B_F5_I1_R	GGCAGAATGCGAATCCGAAC	*CYP51B*	
	CYP51B_F6_Mid_F	TCGCATGATGGAGTGGATGG	*CYP51B*	539
	CYP51B_F6_Mid_R	CTTCGTCCTCTCTCCAGGGA	*CYP51B*	
	CYP51B_F7_I2_F	ATCGTCGCTGGATTCATCGG	*CYP51B*	596
	CYP51B_F7_I2_R	CTCTCAGTTCTCGGATCGCC	*CYP51B*	
	CYP51B_F8-1611 F	AGAAACCGAATGGACAATCCC	*CYP51B*	744
	CYP51B_F8-2354 R	AAGCTCGCTAGTGGTTTATCG	*CYP51B*	
RT-qPCR	Ve-CYP51A-qPCR-S2-1509 F	GCCAGCACTCATCTTCCAG	*CYP51A*	100
	Ve-CYP51A-qPCR-S2-1608 R	CAGACACGAAGCTGTTCCTG	*CYP51A*	
	Ve-CYP51B-qPCR-542 F	TTCACGGATGGACAACAGGG	*CYP51B*	107
	Ve-CYP51B-qPCR-648 R	ATCTTCAATACTCGGGAGGCC	*CYP51B*	
	Ve-Actin-qPCR-S1-92 F	TGCATACGATCCGAGATACCTG	Actin	84
	Ve-Actin-qPCR-S1-175 R	ATTGTTTGGGTGAGCTTGGC	Actin	

**Table 7:** Type III test of fixed effects table generated from statistical analysis in SAS 9.4. “Num DF” indicates the number of degrees of freedom in the model. “Den DF” represents the number of degrees of freedom associates with the model errors. “Pr > F” represents the p-value associated with the F statistic.

Effect	Num DF	Den DF	F Value	Pr > F
**Location**	6	37	6.41	0.0001
**Treatment**	2	186	155.83	<0.0001
**Location Treatment**	12	186	2.58	0.0035
